# Promising agents for chemoprevention of skin cancer

**DOI:** 10.3390/curroncol13050017

**Published:** 2006-10

**Authors:** S.P. Stratton, M.S. Stratton, D.S. Alberts

Skin cancer presents a significant public health problem because of its increasing incidence in the United States, Australia, Northern Europe, and other temperate climates around the world. Yet skin cancer (with the exception of melanoma) gets little press in oncology as compared with other malignancies, presumably because of its relatively low mortality rate. In addition, the morbidity related to skin cancer is often overlooked. Treatment of squamous cell and basal cell carcinomas, by far the two most commonly diagnosed malignancies, is rarely captured by registries.

The latest American Cancer Society data indicate that more than 1 million new non-melanoma skin cancers (nmscs) will be diagnosed in the United States this year 1. Although awareness of skin cancer has been increasing since the mid-1980s (the American Academy of Dermatology now recommends the daily use of sunscreen with a sun protection factor of 15 or greater 2), additional measures are needed to prevent skin cancer. Primary prevention strategies such as sun avoidance and use of sunscreen, among others, have not adequately reduced the incidence of skin cancer.

Precursor lesions indicative of severe sun damage —actinic keratoses—can be treated by topical application of destructive drugs such as 5-fluorouracil, aminolevulinic acid (in photodynamic therapy), diclofenac (a nonsteroidal anti-inflammatory), and imiquimod (a cytokine inducer and immune response modifier). These drugs are effective, but they also may cause painful and irritating local skin toxicities. Less-toxic agents that could be used earlier to prevent age- and sunlight-related precursor lesions would not only prevent treatment-related morbidities, but also would be more cosmetically desirable.

Chronic exposure to ultraviolet (uv) radiation combined with a fair skin type is the most important risk factor for development of skin cancer. Chronic uv exposure also leads to premature aging of the skin and premature appearance of pigmented lesions and wrinkling—two consequences that can command the attention of an image-conscious public. Skin health and related skincare products represent a huge market, and competition for the skincare dollar is fierce.

The cosmetic and skincare industries contain some of the largest multinational conglomerates in the world, in part because the regulatory burden for marketing skincare products is relatively low. Thus, the industry is fraught with bad science and spurious claims. Because of the public interest in skincare and skin health, a tremendous amount of information is broadcast to consumers in mass media such as fashion magazines, health and fitness magazines, television and newspaper advertising, and most recently, the Internet. The real challenge to the consumer is to separate scientifically tested facts from false marketing claims, especially when the message is driven more by the quality of the marketing than of the information content.

## DRUG DEVELOPMENT AND CURRENT RESEARCH

In skin cancer prevention, drug development provides a credible but challenging path to effective chemopreventive products. Clinical testing with appropriate endpoints—for example, quantitative pathology methods such as karyometric analysis 3, protein biomarkers, clinical appearance or regression of neoplastic lesions, progression to cancer, and so on— leading to specific and useful claims for experimental drugs requires an Investigational New Drug application with the U.S. Food and Drug Administration (fda) and the requisite preclinical testing. The scientific diligence required by the fda to successfully obtain an approval lends legitimacy and credibility to the skin cancer chemoprevention research effort. Selection of appropriate drug candidates is crucial, especially given the costs of this “by the book” type of testing.

Much of the current research on skin cancer chemoprevention involves natural products that have been identified based on epidemiologic studies. Epidemiologic findings, along with laboratory and animal studies, have contributed to the identification of molecularly targeted chemopreventive drugs for skin cancer. These drugs are advantageous because their effects are elicited through well-characterized and specific mechanisms of action with potential for a lower side-effect profile. Theoretically, molecularly targeted drugs could provide an ideal skin cancer chemopreventive agent with minimal toxicity and specific action against damaged cells.

An understanding of photobiology will provide the key to successful development of chemoprevention drugs. By causing signature dna mutations, uv radiation is a key contributor to the initiation phase of carcinogenesis. The tumour-promoting effects of uv have been linked to alterations in cellular signal transduction pathways that control the genes that mediate cell proliferation, differentiation, and tumorigenesis. Therefore, factors within this pathway have the potential to serve as ideal molecular targets for a chemopreventive agent 4.

It has also been shown that uv radiation upregulates the local inflammatory response. This response is characterized by accumulation of immune cells within the dermal layer, induction of vascular endothelial adhesion molecules, and production of inflammatory cytokines 5.

Another effect of uv radiation in skin is increased production of prostanoids and upregulation of cyclooxygenase 2 (cox-2) 6. Expression of cox-2 in normal epidermis is low and restricted to regions of differentiated epidermis. In contrast, overexpression of cox-2 in mouse and human skin carcinogenesis contributes to the development of skin cancer 7. These characteristics suggest that factors involved with inflammation, including cox-2, may serve as viable drug targets 8. Several agents involved in these and other uv-regulated signal transduction pathways are under clinical development as potential chemopreventive agents for skin cancer, including retinoids, perillyl alcohol, difluoromethylornithine, green tea polyphenols, cox- 2 inhibitors, resiquimod, and curcumin 9,10.

Another target of interest is the melanocortin 1 receptor. Investigators at the University of Arizona have developed a novel molecule, melanotan, which is one thousand times more potent than α-melanocyte stimulating hormone. Melanotan binds to the receptor and can cause prolonged “natural” tanning to protect the skin from uvb-induced dna damage, even in people with skin types 1 and 2 11.

Clinical trial design in skin cancer chemoprevention is also challenging. Most clinical trials of chemopreventive drugs to date have focused on recurrence of nmsc or on reduction in the number of premalignant skin lesions such as actinic keratoses. The dysplastic nevus, a potential precursor of melanoma, is also a potential target for skin cancer chemoprevention strategies. Premalignant lesions are especially attractive as endpoints because they are more common than frank cancer, resulting in reduced sample size, study length, and cost for clinical trials.

Since the mid-1990s, progress in identifying potential chemopreventive agents and molecular targets for skin cancer has been significant. However, further research is needed to design safe and selective new drugs aimed at these targets for treatment of early skin damage. Development of new drugs that interrupt the pathogenesis of skin cancer may ultimately provide the fastest route to lowering the incidence of this disease.
